# Case Report: Identification of Two Variants of *ALG13* in Families With or Without Seizure and Binocular Strabismus: Phenotypic Spectrum Analysis

**DOI:** 10.3389/fgene.2022.892940

**Published:** 2022-07-11

**Authors:** Tao Cai, Jieting Huang, Xiuwei Ma, Siqi Hu, Lina Zhu, Jinwen Zhu, Zhichun Feng

**Affiliations:** ^1^ Senior Department of Pediatrics, the Seventh Medical Center of PLA General Hospital, Beijing, China; ^2^ Experimental Medicine Section, National Institutes of Health/National Institute of Dental and Craniofacial Research, Bethesda, MD, United States; ^3^ Beijing Key Laboratory of Pediatric Organ Failure, Beijing, China; ^4^ The National Engineering Laboratory for Birth Defects Prevention and Control of Key Technology, Beijing, China; ^5^ Angen Gene Medicine Technology, Beijing, China

**Keywords:** ALG13, mutation, whole-exome sequencing, development delay, X-linked

## Abstract

**Background:** Genetic causes in most affected children with intellectual disability and/or development delay remain unknown.

**Methods:** To identify potential variants responsible for these disorders, we recruited 161 affected families and performed whole-exome sequencing and associated bioinformatics analysis.

**Results:** In the present study, we report the identification of variants in the *ALG13* gene in two of the families. In family 1, a known pathogenic missense variant (c.23T > C; p.V8A) of *ALG13* was identified in a boy and his mother. In family 2, a novel missense variant (c.862C > G; p.L288V) of the same gene was identified in the affected boy and his phenotypically normal mother. Genotype–phenotype correlation analysis by comparing reported 28 different variants (HGMD) showed that three major phenotypes, including various seizures/epilepsy, intellectual disability, and development delay (such as growth, speech, motor, etc.), are present in most affected individuals. However, other phenotypes, such as strabismus and absence of seizure in our second patient, are not reported if any, which may represent a unique case of X-linked recessive nonsyndromic disorder caused by a mutation in *ALG13*.

**Conclusion:** We identified two missense variants in *ALG13* in a cohort of 161 families with affected individuals diagnosed as intellectual disability and/or development delay. A novel c.862C > G mutation may represent a case of X-linked recessive.

## Introduction

The *ALG13* gene encodes a subunit of a bipartite UDP-N-acetylglucosamine transferase that regulates protein folding and stability, which is mapped to Xq23 and widely expressed in human tissues, such as brain, liver, and kidney (MIM: 300776). The first *ALG13* mutation with *de novo* origin was identified in a male infant diagnosed with congenital disorders of glycosylation type I with refractory epilepsy, microcephaly, extrapyramidal, and pyramidal symptoms (Timal, et al., 2012).

Many of the affected individuals were diagnosed with developmental and epileptic encephalopathy 36 (DEE36, MIM: 300884), which is caused by heterozygous or hemizygous mutation in *ALG13*. DEE36 is characterized by the onset of seizures at a mean age of 6.5 months. Most patients present with infantile spasms associated with hypsarrhythmia on EEG, consistent with a clinical diagnosis of West syndrome.

To date, a total of 28 different mutations in *ALG13* (HGMD) have been identified in affected individuals or families with epileptic encephalopathies (Epi, et al., 2013; Moller, et al., 2016), intellectual disability (Bissar-Tadmouri, et al., 2014), infantile spasms (Michaud, et al., 2014), West syndrome (Hino-Fukuyo, et al., 2015) or Lennox–Gastaut syndrome (Zhou, et al., 2018; Stranneheim, et al., 2021), congenital disorder of glycosylation (Alsharhan, et al., 2021), and several rare conditions such as left ventricular obstruction (Jin, et al., 2017) and fetal alcohol syndrome (de la Morena-Barrio, et al., 2018).

In the present study, we report the identification of two variants of *ALG13* from two affected males with development delay and seizures or intellectual disability binocular strabismus, including a novel missense variant (c.862C > G; p.L288V) and a previously reported variant (c.23T > C; p.V8A). For a better understanding of this extremely rare disease, we present a detailed phenotype–genotype correlation analysis and a brief literature review.

## Materials and Methods

### Patients and Standard Protocols

Informed consents were obtained from all participants and in the case of minors, from their parents. This study was approved by the Seventh Medical Center of PLA General Hospital Ethics Committee at Beijing (no. 2022-37). A total of 503 individuals in 161 families, including 175 diagnosed as intellectual disability and/or development delay and 328 unaffected individuals, were recruited for genetic analysis. In the current study, three affected individuals with development defects in two families were presented.

### WES Analysis and Sanger Sequencing

Genomic DNAs were isolated from peripheral blood leukocytes. The captured exome by a SureSelect Human All Exon Kit (Agilent, Santa Clara, CA) was sequenced by HiSeq2000 sequencer (Illumina, San Diego, CA) and analyzed as previously described (Yu, et al., 2016; Zhu, et al., 2019; Li, et al., 2021). The reads were aligned to hg19, and the variants were identified through the GATK pipeline. An average sequence depth of coverage was 149× for exome sequences. Potential pathogenic variants were selected for further bioinformatics analysis. Primers (forward: TCA​CAG​AAG​GCA​GTC​ACT; reverse: CGG​AAT​AAT​GGG​AAG​AGG​AA) were used for Sanger sequencing confirmation of the c.23T > C variant in the ALG13 gene (NM_001099922). Primer sequences for the confirmation of the second variant (c.862C > G) in *ALG13* include the forward ACC​ATA​ATT​GTT​GAG​CTG​AGC​A and reverse TTG​GAT​TCA​ACA​CAG​CTG​GC.

### ALG13 Protein Structure Analysis

The ALG13 protein motif was predicted by the SmartMotif (http://smart.embl-heidelberg.de/). Three-dimensional structure of the AlphaFold ALG13 prediction was obtained from UniProt (https://www.uniprot.org; model ID: AF-Q9NP73-F1). Its associated figures were produced using the program PyMOL (https://pymol.org/2/).

## Results

### Clinical Manifestations of Three Affected Individuals in Two Unrelated Families

**FIGURE 1:**
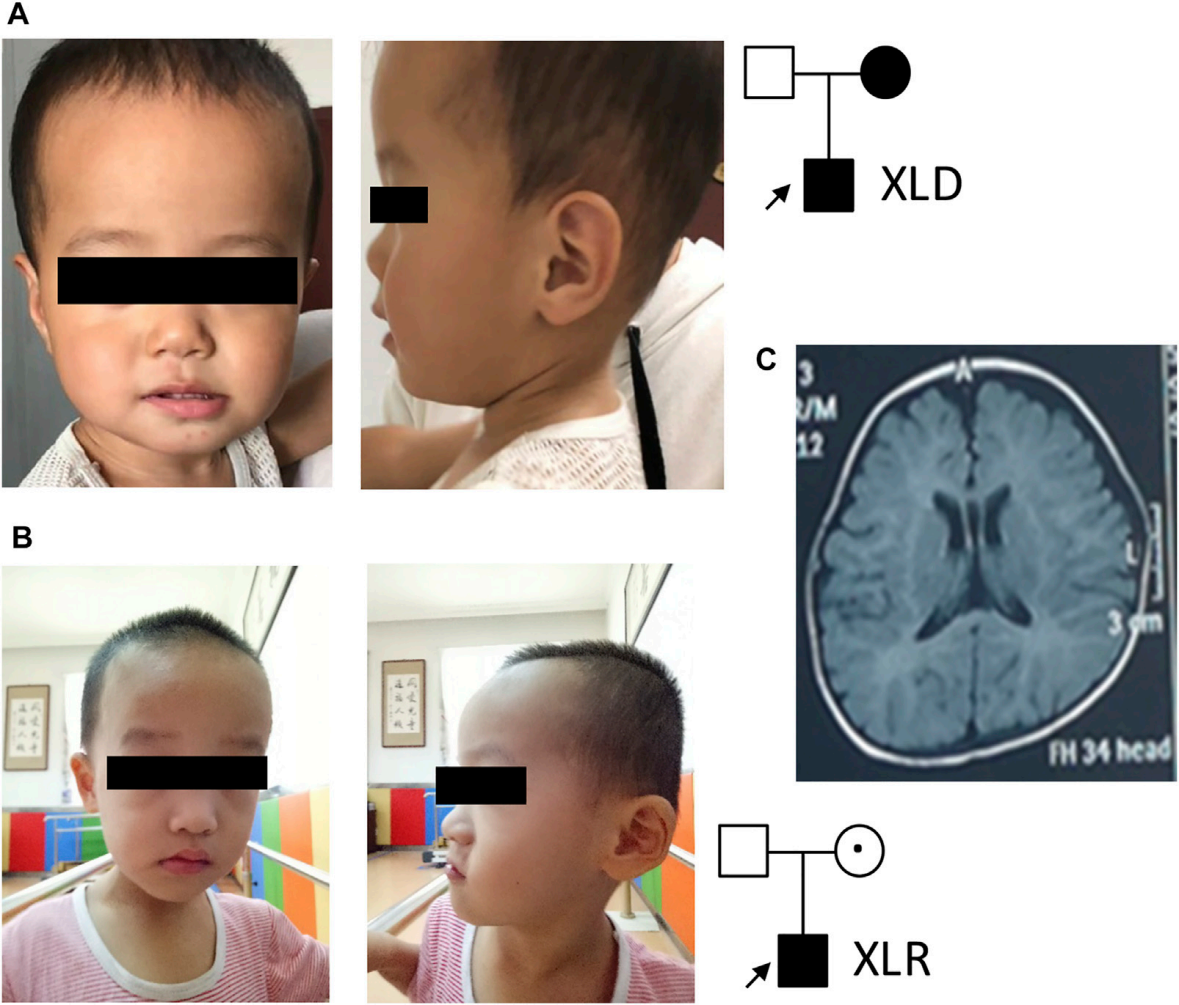
Patient pedigrees and radiographic findings. **(A)** Patient 1 photo and pedigree. **(B)** Patient 2 photo and pedigree. **(C)** Representative MRI image of patient 2.

In family 1, the affected boy (Figure 1A) was first referred to the clinic when he was 2 years old due to intellectual disability, speech and motor development delay, and seizures. Seizures were first observed when the affected boy was 9 months old, which occurred 1–2 times per week and could be controlled by levetiracetam. He could only call “mom” but no other words at 3 years of age. His walking was unstable and could easily fall down. His height and head circumference were in normal ranges. His mother was also diagnosed with intellectual disability, but with no seizures. She could not read or count. His father was normal. An EEK examination at nearly 2 years of age of the body showed abnormal slow-wave activities in bilateral brain. His MRI showed protruding temporal angle of the left lateral ventricle and slightly wider bilateral frontotemporal extracerebral space.

In family 2, a 3-year-old boy (Figure 1B) with intellectual disability and speech and motor development delay was recruited for genetic analysis. His speech was delayed. He could call “mom” and stand up after 2 years of age. His hand movements were not coordinated. His walk was not stable and fell down easily. No seizure was observed. Physical examination showed binocular strabismus and abnormal finger-nose test (FNT). His height and head circumference were in normal ranges. Both of his parents were phenotypically normal. MRI images (Figure 1C) showed delayed myelination and widening of bilateral frontotemporal extracerebral space. His EEK report was normal. A combined analysis of DDST, Gesell, and Bayley tests when he was about 3 years old revealed low levels in his language, social behavior, movement, adaptability, and development quotient.

### Identification of Mutations in the ALG13 Gene by WES

**FIGURE 2:**
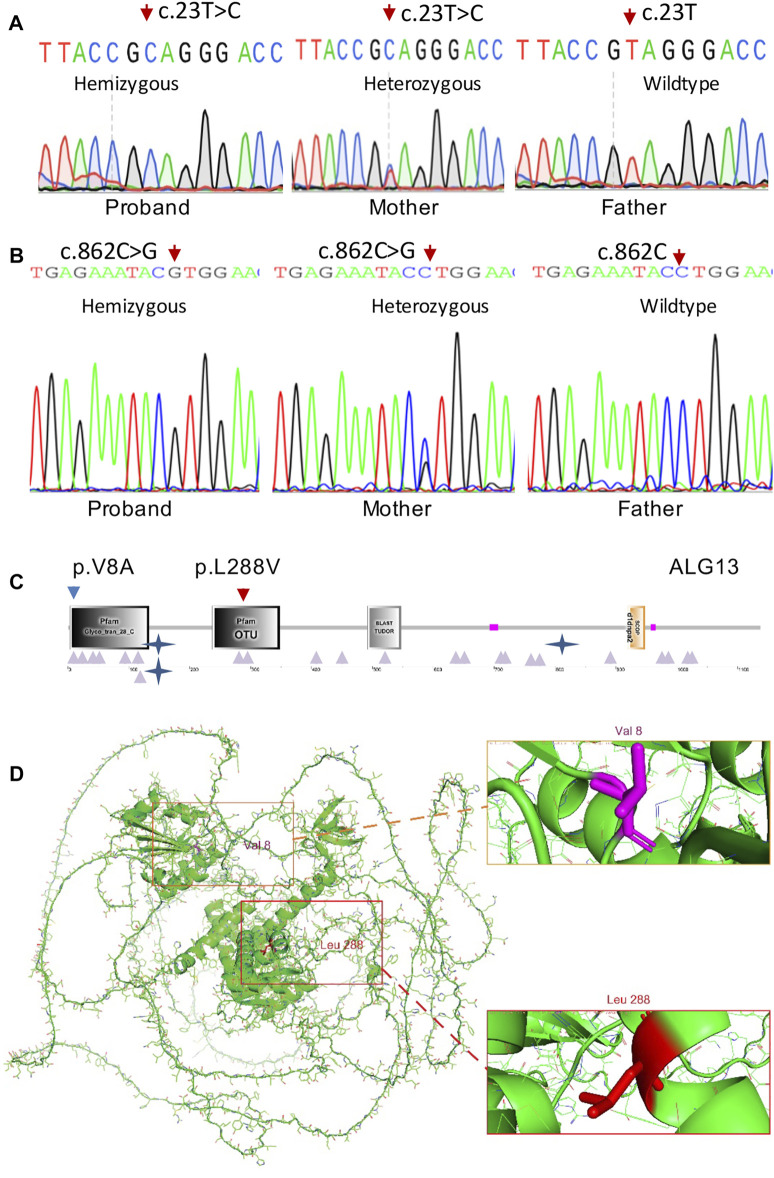
ALG13 mutations and expression. **(A)** Sanger sequencing confirmation of the c.23T > C mutation in case 1 and his mother. **(B)** Sanger sequencing confirmation of the c.862C > G mutation in case 2 and his mother. **(C)** Schematic representations of functional domains of ALG13. The p.V8A mutation of family 1 is located in the Glyco_tans_28 domain at N-terminus (amino acid 3–133). The p.L288V mutation of family 2 is mapped in the OUT domain (amino acid 237–348). **(D)** Residue p.V8 and p.L288 positions are indicated in red in the 3-dimensional structure of the ALG13 protein.

Trio-WES analysis for family 1 identified a known pathogenic missense variant (c.23T > C; p.V8A) in the *ALG13* gene (NM_001099922.3) and further confirmed by Sanger sequencing (Figure 2A) from both the affected boy and his mother. The same mutation as a *de novo* allele was previously detected in a female patient (Datta, et al., 2021), who showed mild developmental delay and seizures starting from the second year of life (Table 1). The p.V8 residue is located in the Glyco_tran_28_C domain (amino acids 3–133) at the N-terminal region of the encoded protein (Figures 2C,D), which involves monogalactosyldiacylglycerol synthase and UDP-N-acetylglucosamine transferase (Pfam, SmartMotif).

**TABLE 1:** Genotype–phenotype correlation analyses for affected individuals with ALG13 variants. The bold presents the phenotype in this study or a special phenotype related to this study.

HGVS	Reported main phenotypes	Brief annotation	References
V8A	Seizures and mild developmental delay (DD)	*De novo* mutation	Datta et al. (2021)
**V8A**	**Seizures, intellectual disability (ID), speech and motor DD**	**Affected mother and son**	This study
I17N	Lennox-Gastaut syndrome	Epileptic encephalopathy	Ji et al. (2019)
E30Q	Microcephaly, global DD, hypoxic ischemic encephalopathy, and hypotonia	OMIM: 300776	Monies et al. (2019)
Q40H	Epileptic encephalopathy		Datta et al. (2021)
T57P	Epileptic encephalopathy		Datta et al. (2021)
K94E	Congenital disorder of glycosylation 1	OMIM: 300884	Timal et al. (2012)
N107S	Lennox-Gastaut syndrome; **strabismus** in patient 3	Epileptic encephalopathy	Epi et al. (2013)
			Paprocka et al. (2021)
N107T	Neurodevelopmental disorder		Geisheker et al. (2017)
G282E	Epileptic encephalopathy, infantile		Wei et al. (2018)
**L288V**	**ID, speech and motor DD; no seizure; finger-nose test (FNT, +); strabismus; MRI: myelination delayed, et al**	EEK (-); the variant-carrier mother is normal	This study
P294S	West syndrome and optic nerve atrophy	Infantile spasms	Hino-Fukuyo et al. (2015)
K411N	Neurological disorder	No detailed info	Jiao et al. (2019)
E463G	Seizures, motor, and speech DD		Gadomski et al. (2017)
Q547H	Epileptic encephalopathy		Moller et al. (2016)
E640K	ID, gross and fine motor DD		Monies et al. (2019)
P658L	Lennox-Gastaut syndrome	Epileptic encephalopathy	Stranneheim et al. (2021)
R701H	Left ventricular obstruction and neurodevelopmental disorder		Jin et al. (2017)
S702R	Seizures and ID		Farwell et al. (2015)
V758F	Congenital disorder of glycosylation	OMIM: 300884	Alsharhan et al. (2021)
R769W	Epilepsy, motor DD, and learning disability		Monies et al. (2019)
S891F	Fetal alcohol syndrome, predisposition to		de la Morena-Barrio et al. (2018)
P963S	Epilepsy and neurodevelopmental abnormalities		Amadori et al. (2020)
G972V	Congenital disorder of glycosylation	OMIM: 300884	Alsharhan et al. (2021)
P1005S	Congenital disorder of glycosylation	OMIM: 300884	Alsharhan et al. (2021)
P1073R	ID, X-linked		Harripaul et al. (2018)
Y1074C	ID, nonsyndromic		Bissar-Tadmouri et al. (2014)
c.384-5C > T	Lennox-Gastaut syndrome	A triad of seizure, EEG findings, and ID	Zhou et al. (2018)
c.384-1G > A	Epilepsy		Hesse et al. (2018)
S820_P862del	Congenital disorder of glycosylation	OMIM: 300884	Alsharhan et al. (2021)
Del 9p24.2	Infantile spasms		Michaud et al. (2014)

*Intellectual disability (ID); development delay (DD).

Trio-WES analysis for family 2 identified a previously undescribed potentially pathogenic missense variant (c.862C > G) from the affected boy and his phenotypically normal mother. Sanger sequencing further confirmed this variant (Figure 2B). Bioinformatics analysis revealed that this variant is not present in ExAC or the in-house database and predicted to be disease-causing by MutationTaster and Polyphen2. Smart Motif analysis revealed that the mutation p.L288 is mapped to the OTU domain (amino acids 237–348) in the ALG13 protein (Figures 2C,D), which is OTU-like cysteine protease motif (Makarova, et al., 2000).

### Genotype and Phenotype Profile Related to *ALG13* Mutations

As shown in Table 1, we summarized 28 different mutations that are listed in HGMD and two mutations identified in the present study. Affected individuals and/or families were either in X-linked dominant (XLD) or X-linked recessive (XLR) pattern or with *de novo* mutation (DNM) origins. Family 1 in our case is in the XLD form, while family 2 is in the XLR pattern. Most of the mutations in Table 1 are missense variants (27/30); three of them are splicing and deletion mutations (3/30). Three major phenotypes, including various seizures/epilepsy, intellectual disability, and development delay (such as growth, speech, motor, etc.), are observed in most of the cases. Less frequently observed phenotypes include strabismus, optic nerve atrophy, left ventricular obstruction, and ataxia.

## Discussion

In the present study, we identified two variants in the *ALG13* gene in patients with either typical phenotypes in family 1 with XLD inheritance form (seizures, intellectual disability, speech, and motor development delay) or atypical phenotypes in family 2 with XLR inheritance pattern (mild intellectual disability, speech and motor development delay, mild ataxia, and binocular strabismus, but no seizures). Previously, only 28 different variants were reported (HGMD). Three of them (c.845G > A; p.G282E, c.1233G > C; p.K411N and c.384-5C > T) were identified in Chinese populations (Wei, et al., 2018; Zhou, et al., 2018; Jiao, et al., 2019). Our findings expanded the *ALG-13*-related mutation spectrum and *ALG-13*-associated clinic manifestations.

Genotype–phenotype correlation analysis indicates that the mild clinical manifestations of the patient in family 2 is resulted from a mild pathogenic mutation (c.862C > G; p.L288V). In fact, both leucine and valine in the p.L288V allele are alpha-amino acids, which implies that they contain an alpha-amino group, an alpha-carboxylic acid group, and a side chain isobutyl group/isopropyl group. Based on the AlphaFold predicted structure model (Figure 2D), the Leu288 residue is located in α-helix.

In contrast, alanine in the p.V8A variant in family 1 is a simple amino acid, which has just a methyl as its side chain. Based on the AlphaFold predicted structure model (Figure 2D), Val8 residue is located in the loop region involving glycosyltransferase activity (amino acids 1–125), thereby causing more severe clinic phenotypes as we described earlier.

Based on several commonly used gene expression databases, such as BioGPS and human brain transcriptome, the human *ALG13* gene is widely expressed in many tissues, including neurons in developing and adult brain tissues (Supplementary Figure S1). Brain-associated clinical manifestations, such as seizures and intellectual disability, are apparently correlated with cortical and central nervous dysfunctions in the affected individuals with *ALG13* variants. Additional rare phenotypes, such as ataxia, nystagmus, and strabismus, are potentially associated with developmental defects or dysfunctions of the cerebellum and brain stem tissues.

In the *Alg13* knockout mouse model, *Alg13* deficiency resulted in an increased seizure and susceptibility in the *Alg13* ^−/−^ mice (Gao, et al., 2019). Previous studies also explored the possible mechanisms of *Alg13*-involved epilepsy by showing hyperactive mTOR signaling pathways in the cortex and hippocampus of *Alg13* ^−/−^ mice (Gao, et al., 2019; Huo, et al., 2020). Further studies using patch-clamp recordings demonstrated that *Alg13* ^−/−^ mice show a marked decrease in the gamma-aminobutyric acid A receptor (GABAAR)–mediated inhibitory synaptic transmission (Huo, et al., 2020). At the human level, a majority of variants are missense, which are linked to either X-linked dominant phenotypes due to stronger pathogenic variants (such as the variant in family 1) or X-linked recessive phenotypes due to mild pathogenetic variants (such as the variant in family 2).

## Conclusion

Taken together, we provided clinical and bioinformatics evidences that two *ALG13* variants are pathogenic for the affected individuals with *ALG13*-associated phenotypes. However, the underlying mechanism remains to be explored in further studies.

## Data Availability

The original datasets presented in the study are included in the article/Supplementary Material, further inquiries can be directed to the corresponding author.
