# Supplementary material for: Case Report: Identification of Two Variants of ALG13 in Families With or Without Seizure and Binocular Strabismus: Phenotypic Spectrum Analysis
Source: Front Genet. 2022 Jul 11;13:892940. doi: 10.3389/fgene.2022.892940 (PMC9310169; doi:10.3389/fgene.2022.892940)
Supplement: Supplementary file 1 [file Presentation1.PPTX]

## Slide 1
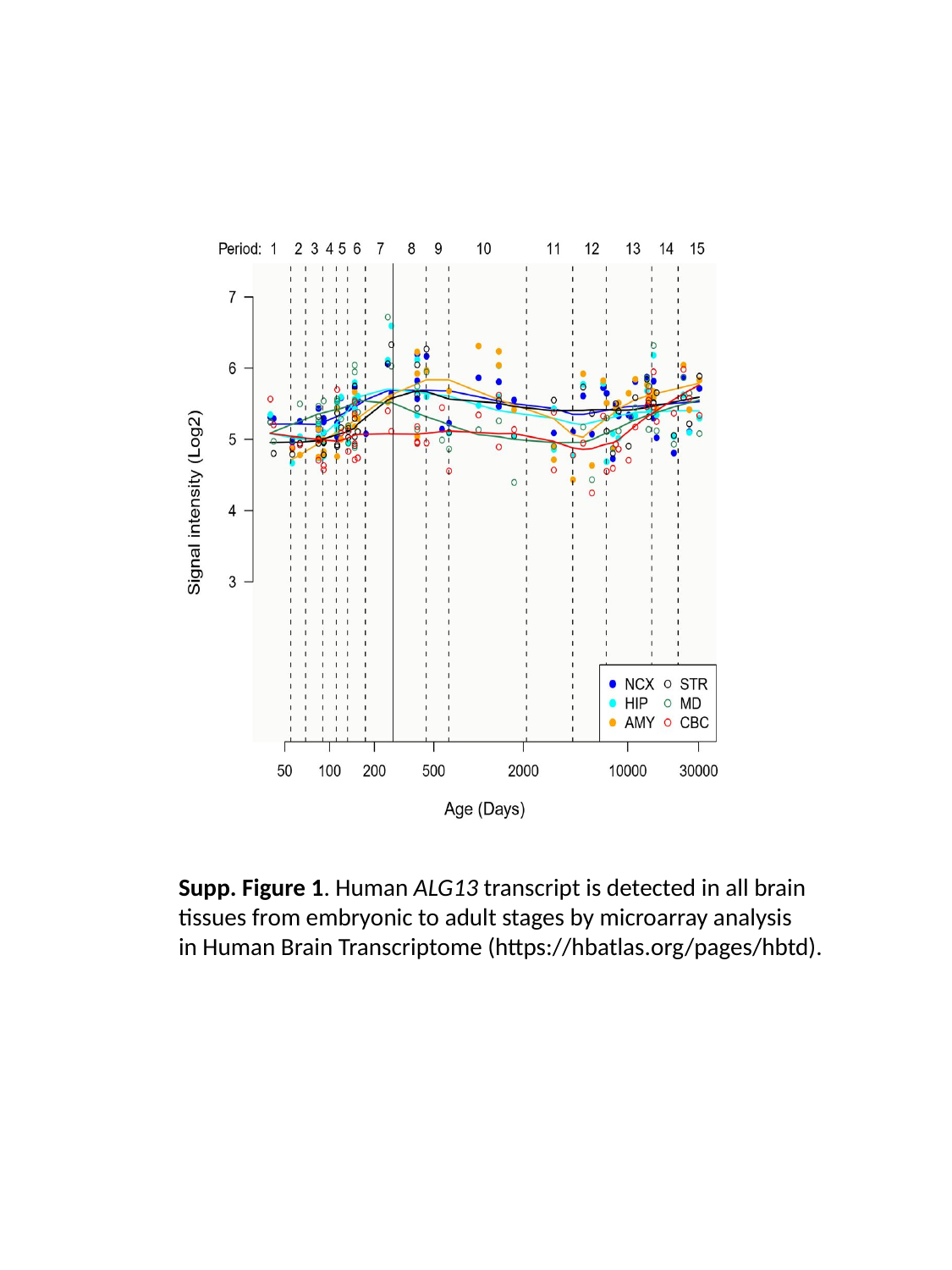

Supp. Figure 1. Human ALG13 transcript is detected in all brain tissues from embryonic to adult stages by microarray analysis
in Human Brain Transcriptome (https://hbatlas.org/pages/hbtd).
